# Racial and ethnic disparities in viral acute respiratory infections in the United States: protocol of a systematic review

**DOI:** 10.1186/s13643-021-01749-8

**Published:** 2021-07-02

**Authors:** Neia Prata Menezes, Jowanna Malone, Carrie Lyons, Kechna Cadet, Lorraine Dean, Gregorio Millett, Stefan Baral

**Affiliations:** 1 Department of Epidemiology, Bloomberg School of Public Health, Johns Hopkins University, Baltimore, MD USA; 2 Department of Mental Health, Bloomberg School of Public Health, Johns Hopkins University, Baltimore, MD USA; 3 Public Policy Office, AmfAR, Foundation for AIDS Research, Washington, DC USA; 4 Center for Public Health and Human Rights, Bloomberg School of Public Health, Johns Hopkins University, Baltimore, MD USA

**Keywords:** Acute respiratory infection, Racial disparities, Health disparities, Influenza-like illness, Pandemic preparedness, Pandemic response, Structural racism

## Abstract

**Background:**

The COVID-19 pandemic caused by SARS-CoV-2 has highlighted consistent inequities in incidence, disease severity, and mortality across racial and ethnic minority populations in the United States (US) and beyond. While similar patterns have been observed with previous viral respiratory pathogens, to date, no systematic review has comprehensively documented these disparities or potential contributing factors. In response, this review aims to synthesize data on racial and ethnic disparities in morbidity and mortality due to viral acute respiratory infections (ARI) other than SARS-CoV-2. This review will focus on understanding structural health and social factors to contextualize race and ethnicity driving these disparities in the US.

**Methods:**

We will conduct a systematic review of studies published from January 1, 2002, onward. Our search will include PubMed/MEDLINE, EBSCO Host-CINAHL Plus, PsycInfo, EMBASE, and Cochrane Library databases to identify relevant articles. We will include studies of any design that describe racial/ethnic disparities associated with viral ARI conducted in the US. Primary outcomes include incidence, disease severity or complication, hospitalization, or death attributed to ARI. Secondary outcomes include uptake of preventive interventions including vaccination, handwashing, social distancing, and wearing masks. Two reviewers will independently screen all citations, full-text articles, and abstract relevant data. Data characterizing individual-, community-, and structural-level factors associated with these disparities will be abstracted to better understand the underlying structural inequities contributing to racial disparities in ARI. We will assess the methodological quality of all studies and will conduct meta-analyses using random effects models if appropriate.

**Discussion:**

Findings from this systematic review will shed light on patterns of racial and ethnic disparities in viral ARI in the United States to support mathematical modeling of epidemic trajectories, intervention impact, and structural drivers of transmission, including structural racism. Moreover, data emerging from this review may reignite pandemic preparedness focused on communities with specific vulnerabilities related to living and working conditions given prevailing structural inequities, thus facilitating improved future pandemic responses to novel or endemic viral respiratory pathogens.

**Systematic review registration:**

PROSPERO CRD42020219771

**Supplementary Information:**

The online version contains supplementary material available at 10.1186/s13643-021-01749-8.

## Background

Emerging evidence has highlighted the stark racial and ethnic disparities in the risk of infection and death caused by the novel severe acute respiratory syndrome coronavirus 2 (SARS-CoV-2), the virus responsible for COVID-19. In the United States, African American, Latinx, and indigenous populations have been disproportionately affected by COVID-19-related infections and associated morbidity and mortality [1–4]. Notably, these disparities have mirrored trends observed in prior epidemics of other viral acute respiratory infections (ARI). During the 1918 influenza pandemic, Black Americans were more likely to die compared to their white counterparts [5]. Most recently, during the 2009 H1N1 influenza pandemic, minority groups were more likely to be hospitalized and to die compared to non-Hispanic white populations [6, 7]. The purpose of this systematic review is to investigate racial and ethnic disparities in morbidity and mortality of prior outbreaks, epidemics, and pandemics of ARI to provide context in understanding the disparate impact of the current COVID-19 epidemic across the United States.

Despite the growing interest to explore literature concerning racial disparities and social determinants of ARI, there are no systematic reviews comprehensively characterizing research focused on racial and ethnic disparities from former infectious disease outbreaks of viral respiratory pathogens [8]. Aside from recent literature concerning SARS-CoV-2, there is a lack of research documenting racial disparities in prior outbreaks or pandemics. However, there is existing evidence of racial disparities involving ARI. In the United States, research has shown that Black children have higher rates of hospitalizations from ARI compared to white children, although they do not differ in severity of disease once hospitalized [9]. There has also been evidence that acute infections can contribute to racial disparities in stroke-related deaths; however, this included non-respiratory tract infections in addition to ARI [10].

Considering the ever-growing focus on the contribution of racial inequities to multiple facets of health, and the present burden of COVID-19-related deaths on African-American, Latinx, and indigenous communities in the current pandemic, it is imperative to understand factors, including structural racism, that have contributed to these disparities in earlier outbreaks. Such knowledge would inform development of a pandemic preparedness response that ensures protection of the most vulnerable.

Framing pandemic preparedness through a social justice lens is not a novel concept and has been previously emphasized as integral to mounting an effective response strategy [11–13]. Nevertheless, underscoring the protection of groups at highest risk of disease burden based on socioeconomic factors defining living and working conditions has yet to be incorporated into existing national strategies [14]. By failing to protect the most vulnerable, we not only miss the opportunity to prevent and mitigate disease burden among those at highest risk and in the population as a whole, but also inadvertently perpetuate the systemic inequities that contribute to ongoing health disparities in minority communities. A deepened understanding of the scale and drivers of racial disparities in ARI attributable to structural and systemic inequities will not only prevent further disparities from occurring in the event of future outbreaks or pandemics but can also alleviate present disparities in endemic ARI.

To address the gaps in the literature, the aim of this systematic review is to synthesize the racial and ethnic disparities in the incidence, morbidity, and mortality of viral acute respiratory infections from 2002 onward in the United States. This review also aims to determine which racial/ethnic populations are disproportionately affected and evaluate underlying drivers of these disparities including structural racism. We will also describe the individual-, community-, and structural-level factors correlated with race/ethnicity, including social determinants of health such as socio-economic status, geography/neighborhood, healthcare accessibility, which are associated with increased morbidity and mortality in acute respiratory infections.

## Methods

### Protocol registration and reporting

This protocol is registered under the PROSPERO database (CRD42020219771) and is being reported in accordance with the recommendations specified in the Preferred Reporting Items for Systematic Reviews and Meta-analyses Protocols (PRISMA-P) (see Additional file 1) [15]. The planned review will be reported using the PRISMA 2020 statement and any amendments to the protocol will be documented in the final review [16].

### Eligibility criteria

#### Population

The population will include persons of any age and gender who are at risk for, or infected with, a respiratory viral pathogen (influenza virus, respiratory syncytial virus (RSV), Middle East respiratory syndrome (MERS), severe acute respiratory syndrome coronavirus 1 (SARS), parainfluenza virus, measles virus, rubella virus, rhinovirus, and/or adenovirus). We will exclude study participants outside of the United States. Age, ethnicity, gender, and other relevant information will be disaggregated if appropriate.

#### Types of studies to be included

This systematic review will include any primary studies of any design (quantitative or qualitative) that describe racial/ethnic disparities associated with the viral respiratory pathogens listed above. We will include both quantitative observational (such as cohort, case-control, and cross-sectional studies) and qualitative studies. Using qualitative studies is a best practice in uncovering new information where little research exists, or where quantitative studies are unable to provide insight into the complex interplay between variables [17]. Thus, we will use qualitative data to understand the reasons behind how and why individual-, community- and structural-level factors (e.g., structural racism) are experienced to further elucidate the disparate effects of these factors on racial and ethnic minority groups. This review will restrict studies that are publications in peer-reviewed journals or abstracts at conferences with peer-reviewed blinded abstract selection processes. The review will also include studies that, at a minimum, capture and report information on racial/ethnic disparities in ARI and list details regarding data sampling techniques. We will further restrict our search to articles conducted in English, based in the United States, published since January 1, 2002, with data collection that started no later than January 1, 2000. Given that the first case of SARS was detected in late 2002, we will include all articles published from January 2002 onward [18].

#### Comparison

Studies will measure the following as an exposure: non-white race/ethnicity, such as Black/African American, Asian, American Indian/Alaska Native, Native Hawaiian/other Pacific Islander, non-white Hispanic, or Latinx, as defined by the United States Census Bureau [19]. All studies will be included that compare non-white race/ethnicity to the following: no comparison, white race/ethnicity, and other non-white race/ethnicity.

#### Outcomes to be measured

Based on literature regarding racial and ethnic disparities in ARI-associated morbidity and mortality, the primary outcomes will be ARI incidence, disease severity/complication due to ARI, hospitalization due to ARI, and death attributed to ARI [8]. Secondary outcomes of this review will assess uptake of preventive interventions shown to be effective for mitigating the spread of viral respiratory pathogens which include vaccine coverage and uptake, handwashing, social distancing, and wearing masks [20]. Given the importance of these interventions for halting the spread of viral respiratory pathogens, we will determine whether racial/ethnic disparities exist in the delivery to and uptake and adherence of these interventions. We will report measured effects comparing differences between racial and ethnic groups for any of the primary and secondary outcomes outlined.

#### Exclusion criteria

This review will exclude articles that only address disparities with the novel SARS-CoV-2 that causes COVID-19, were conducted outside of the United States, were conducted among non-human, animal subjects, have a sample size less than 100 persons, do not mention race or ethnicity in the abstract, and/or are based on modeling data.

### Information sources and search strategies

In partnership with an information specialist at Johns Hopkins University, we will search the following electronic databases (from January 2002 onward): the National Library of Medicine’s MEDLINE database using the PubMED interface, EBSCO Host- CINAHL Plus, PsycInfo, EMBASE, and Cochrane Library. Search criteria and terms were created based on validated peer-reviewed systematic reviews and articles regarding racial disparities and acute respiratory infections [8]. We will search the gray literature by hand. Reference lists of identified articles and reports will be reviewed for additional articles.

We piloted multiple search strategies to optimize an approach that is highly sensitive yet prioritizes identifying relevant articles. The search strategies are comprised of a combination of controlled MeSH terms and other search terms that cover two independent concepts: acute respiratory infections and racial/ethnic disparities (see Additional file 2). Search terms pertaining to acute respiratory infections include terms relating to viral infections due to influenza, other coronaviruses (excluding SARS-CoV-2), other viral respiratory pathogens, and general terms for acute respiratory infection and/or influenza-like illness. Terms associated with racial disparities include terms relating to general health disparities or inequities, race/ethnicity, socioeconomic factors (e.g., poverty, education), and historically marginalized communities (undocumented immigrants, incarcerated persons). We opted not to include search terms specific to racial/ethnic groups to mitigate the potential for missing articles due to improper indexing. Additionally, by including terms that are not limited to racial/ethnic groups (e.g., poverty, health care access), we hope to capture information on disparities associated with structural and systemic inequities, including structural racism, that overwhelmingly affect racial and ethnic minority communities in the United States.

### Screening and selection process

The literature review consists of an iterative title review, abstract review, and full-text review for articles that meet the criteria [21]. Two reviewers will conduct parallel screening of titles found in the search. If either one or both of the two reviewers selects a title to move forward to abstract review, the abstract will be reviewed independently and assessed for inclusion in the full article review. If either one or both of reviewers selects the abstract for full article review, the article will be selected for full article review. If at the full article review there is a disagreement between the first two reviewers regarding data extraction, a third reviewer will review and solve the disagreement.

### Risk of bias and quality assessment of individual studies

All observational studies selected for review will be assessed for risk of bias using the Newcastle-Ottawa Scale, a quality assessment tool for non-randomized studies that assesses articles according to three components: study group selection, group comparability, and outcome ascertainment [22]. Risk of bias will be assessed both at the study and outcome levels for quantitative studies.

### Data extraction

Data will be extracted for each full article reviewed and entered into a pre-piloted data collection form based on COVIDENCE—a commercially available web-based tool for conducting and managing systematic reviews. Reviewers will be trained to abstract the relevant data from the articles using the data abstraction tool.

The following fields will be abstracted from all included studies:

- Source reference
- Source type (e.g., journal article, abstract) and publication year
- Population (adult ≥18, child<18, and any qualifying characteristics such as race/ethnicity, sex/gender, etc.)
- Geographic setting (within the United States)
- Sample size
- Nature of study (descriptive, quantitative, qualitative)
- Study design (e.g., cross-sectional, etc.)
- Individual-, community-level, or structural-level factors evaluated
- Type of individual-, community-, or structural-level factor discussed (e.g., race/ethnicity, socioeconomic status, healthcare coverage, housing, citizenship status, neighborhood location, food security)
- Measures of effect for all primary or secondary outcomes of interest, including proportions, relative risks, odds ratios, or hazard ratios if time series data available
- Summary of author interpretations/conclusions

### Data synthesis

Given the inclusion of both quantitative and qualitative studies, we anticipate that articles may differ in their statistical and methodological approaches. For qualitative findings, we will synthesize primary studies to explore heterogeneity descriptively rather than statistically, such as through structured narratives or summary tables. If race/ethnicity exposures are similarly measured, we will assess consistency of thematic results. The “Enhancing transparency in reporting the synthesis of qualitative research” (ENTREQ) 21-item checklist groups findings into five main domains: introduction, methods and methodology, literature search and selection, appraisal, and synthesis of findings [23]. Using the ENTREQ guidelines, we will construct evidence tables to provide a description of included studies, using data from each paper (e.g., study characteristics, context, participants, outcomes, and findings).

For quantitative studies, reporting the same outcome, among populations deemed sufficiently similar, we will conduct meta-analysis using random effects meta-analytic models. We aim to assess heterogeneity and a random intercepts model will be used to account for between-study variation. To determine the appropriateness of conducting a meta-analysis, we will evaluate heterogeneity within selected studies by examining forest plots and conducting Cochrane’s Q and I^2^ statistical tests. If the included studies do not yield pooled estimates, we will instead conduct a narrative synthesis using the “Synthesis without meta-analysis” (SWiM) in systematic reviews guidelines, a 9-item checklist [24]. We will synthesize key elements of the methods (e.g., describe groupings of studies for synthesis, methods used, any criteria applied to prioritize results for synthesis, certainty of evidence), results (e.g., provide a description of the synthesized findings and the certainty of the findings), and discussions (e.g., report the limitations of the synthesis methods used and/or the groupings used in the synthesis).

## Confidence in cumulative evidence

For quantitative studies, we will assess the quality of the body of evidence contributing to the pooled effect estimate for each outcome using criteria recommended by the Grading of Recommendations Assessment, Development and Evaluation (GRADE) Working Group: GRADE evidence certainty for individual outcomes [25–30]. We will assess the quality of evidence across the following domains: risk of bias, consistency, directness, precision, and publication bias. We may consider additional domains if appropriate. Funnel plots will be used to assess for risk of publication bias across individual studies where 10 or more similar studies are included. If study protocols are available, we will compare the outcomes reported in the protocol to those in the published report to assess for selective outcome reporting. If protocols are not available, we will compare the outcomes reported in the methods and results sections of the published study. Finally, we will compare the fixed effect estimate to the random effects model to determine whether small sample bias exists.

## Discussion

The aim of this review is to investigate disparities in morbidity and mortality in acute respiratory viral infections other than SARS-CoV-2. Some practical challenges are expected in the identification of existing evidence. First, although we are focusing our search on racial and ethnic disparities in ARI outcomes, our search strategy will not filter out articles based on specific race or ethnicity terms from the title screening, as these terms may not be present. Instead, we will assess for the presence of racial and ethnic comparisons during the abstract screening phase. Furthermore, the medical library indexing of articles may influence the results obtained in this review. It is common practice to report outcomes by race; however, if differential outcomes are not a primary objective of the study, the article may not be indexed in the medical library accordingly and therefore may not be represented in this review, or it will be excluded. If we are unable to identify any relevant articles, we will consider expanding our search terms and will document any changes to the protocol in PROSPERO and the final manuscript of the review, which is intended for publication.

Second, racial and ethnic disparities are documented to be associated with interrelated socioeconomic factors representing macrolevel inequalities such as income, neighborhood, stigma and discrimination, and barriers or access to healthcare [11]. Given this, studies may focus on upstream determinants of racial disparities, or manifestations of structural inequalities other than race. The search protocol has been developed to consider this complexity; however discerning intersecting factors is an expected challenge of this review. Lastly, under-representation of racial and ethnic minorities in clinical trials has been documented in other contexts [31]. Specifically, historical ethics violations of human subjects’ research have disproportionately affected minorities in the United States and potentially deterred study participants from minority communities. Therefore, study populations may not be representative of racial and ethnic minorities. These potential challenges may limit the comprehensiveness of this review and therefore limit the conclusions.

The findings from this systematic review will provide context and insight to understand patterns of disparity in viral acute respiratory infections in the United States. Furthermore, this review may support a robust comprehension of the manifestations of structural and social inequities affecting racial and ethnic minorities and their influence on ARI outcomes. Leveraging this information could influence development and implementation of studies on the differential impact of COVID-19 across the United States. Moreover, this information could be used to inform the development of resource-based strategies facilitating the prevention of both acquisition and onward transmission to alleviate the structural inequities driving racial and ethnic disparities prevalent in ARI. Finally, the Biden-Harris Administration have proposed the establishment of a COVID-19 Racial and Ethnic Disparities Task Force as an integral component of their plans to address the ongoing COVID-19 pandemic and pandemic preparedness moving forward [32]. We expect that that the results of this systematic review adds to the body of evidence that will drive future decision-making of this task force by reigniting a focus on pandemic preparedness and public health responses to other ARI that underscores the anticipation of disparities and the implementation of equitable and human rights affirming programs to mitigate them.

## Supplementary Information

**Additional file 1:** PRISMA-P+checklist.

**Additional file 2:** MEDLINE search terms.

## Data Availability

Not applicable. All data will be made available upon the completion of the review process.

## Abbreviations

ARI: Acute respiratory infection
ILI: Influenza-like illness
RSV: Respiratory syncytial virus
MERS: Middle East respiratory syndrome
SARS: Severe acute respiratory syndrome
SARS-CoV-2: Severe acute respiratory syndrome coronavirus 2
COVID-19: Coronavirus disease 2019

## References

[1] Vahidy FS, Nicolas JC, Meeks JR, Khan O, Jones SL, Masud F, et al. Racial and ethnic disparities in SARS-CoV-2 pandemic: analysis of a COVID-19 observational registry for a diverse US metropolitan population. medRxiv. 2020. 10.1136/bmjopen-2020-039849 PMC7418666 32784264

[2] Rodriguez-Diaz CE, Guilamo-Ramos V, Mena L, Hall E, Honermann B, Crowley JS (2020). Risk for COVID-19 infection and death among Latinos in the United States: examining heterogeneity in transmission dynamics. Ann Epidemiol.

[3] Poteat T, Millett G, Nelson LE, Beyrer C (2020). Understanding COVID-19 risks and vulnerabilities among Black communities in America: the lethal force of syndemics. Ann Epidemiol.

[4] Cases of Coronavirus Disease (COVID-19) in the U.S.: COVID Data Tracker: Centers for Disease Control and Prevention; [Available from: https://covid.cdc.gov/covid-data-tracker/?CDC_AA_refVal=https%3A%2F%2Fwww.cdc.gov%2Fcoronavirus%2F2019-ncov%2Fcases-updates%2Fcases-in-us.html#cases_casesper100klast7days.

[5] Økland H, Mamelund S-E (2019). Race and 1918 influenza pandemic in the united states: a review of the literature. Int J Environ Res Public Health.

[6] Information on 2009 H1N1 impact by race and ethnicity: Centers for Disease Control and Prevention; [Available from: http://www.cdc.gov/h1n1flu/race_ethnicity_qa.htm.

[7] Castrodale L (2009). Deaths Related to 2009 Pandemic Influenza A (H1N1) Among American Indian/Alaska Natives---12 States, 2009.

[8] Moran E, Kubale J, Noppert G, Malosh RE, Zelner JL. Inequality in acute respiratory infection outcomes in the United States: a review of the literature and its implications for public health policy and practice. medRxiv. 2020.

[9] Iwane MK, Chaves SS, Szilagyi PG, Edwards KM, Hall CB, Staat MA, Brown CJ, Griffin MR, Weinberg GA, Poehling KA, Prill MM, Williams JV, Bridges CB (2013). Disparities between black and white children in hospitalizations associated with acute respiratory illness and laboratory-confirmed influenza and respiratory syncytial virus in 3 US counties—2002–2009. Am J Epidemiol.

[10] Levine DA, Langa KM, Rogers MA (2014). Acute infection contributes to racial disparities in stroke mortality. Neurology.

[11] DeBruin D, Liaschenko J, Marshall MF (2012). Social justice in pandemic preparedness. Am J Public Health.

[12] Hutchins SS, Truman BI, Merlin TL, Redd SC (2009). Protecting vulnerable populations from pandemic influenza in the United States: a strategic imperative. Am J Public Health.

[13] Hutchins SS, Fiscella K, Levine RS, Ompad DC, McDonald M (2009). Protection of racial/ethnic minority populations during an influenza pandemic. Am J Public Health.

[14] Health USDo, Services H (2005). HHS pandemic influenza plan: US Department of Health and Human Services.

[15] Moher D, Shamseer L, Clarke M, Ghersi D, Liberati A, Petticrew M (2015). Preferred reporting items for systematic review and meta-analysis protocols (PRISMA-P) 2015 statement. Systematic reviews.

[16] Page M, McKenzie J, Bossuyt P, Boutron I, Hoffmann T, Mulrow C (2021). The PRISMA 2020 statement: an updated guideline for reporting systematic reviews. BMJ.

[17] Ashing-Giwa KT (2005). Can a culturally responsive model for research design bring us closer to addressing participation disparities? Lessons learned from cancer survivorship studies. Ethn Dis.

[18] Cherry JD (2004). The chronology of the 2002–2003 SARS mini pandemic. Paediatr Respir Rev.

[19] About Race: United States Census Bureau (USC); 2020 [Available from: https://www.census.gov/topics/population/race/about.html.

[20] Larson EL (2007). Warned, but not well armed: preventing viral upper respiratory infections in households. Public Health Nurs.

[21] Mateen F, Oh J, Tergas A, Bhayani N, Kamdar B (2013). Titles versus titles and abstracts for initial screening of articles for systematic reviews. Clin Epidemiol.

[22] Peterson J, Welch V, Losos M, Tugwell P (2011). The Newcastle-Ottawa scale (NOS) for assessing the quality of nonrandomised studies in meta-analyses.

[23] Tong A, Flemming K, McInnes E, Oliver S, Craig J (2012). Enhancing transparency in reporting the synthesis of qualitative research: ENTREQ. BMC Med Res Methodol.

[24] Campbell M, McKenzie JE, Sowden A, Katikireddi SV, Brennan SE, Ellis S, et al. Synthesis without meta-analysis (SWiM) in systematic reviews: reporting guideline. bmj. 2020;368. 10.1136/bmj.l6890 PMC7190266 31948937

[25] Guyatt GH, Oxman AD, Kunz R, Brozek J, Alonso-Coello P, Rind D, Devereaux PJ, Montori VM, Freyschuss B, Vist G, Jaeschke R, Williams JW, Murad MH, Sinclair D, Falck-Ytter Y, Meerpohl J, Whittington C, Thorlund K, Andrews J, Schünemann HJ (2011). GRADE guidelines 6. Rating the quality of evidence—imprecision. J Clin Epidemiol.

[26] Guyatt G, Oxman AD, Akl EA, Kunz R, Vist G, Brozek J, Norris S, Falck-Ytter Y, Glasziou P, deBeer H (2011). GRADE guidelines: 1. Introduction—GRADE evidence profiles and summary of findings tables. J Clin Epidemiol.

[27] Guyatt GH, Oxman AD, Vist G, Kunz R, Brozek J, Alonso-Coello P, Montori V, Akl EA, Djulbegovic B, Falck-Ytter Y, Norris SL, Williams JW Jr, Atkins D, Meerpohl J, Schünemann HJ (2011). GRADE guidelines: 4. Rating the quality of evidence—study limitations (risk of bias). J Clin Epidemiol.

[28] Guyatt GH, Oxman AD, Montori V, Vist G, Kunz R, Brozek J, Alonso-Coello P, Djulbegovic B, Atkins D, Falck-Ytter Y, Williams JW, Meerpohl J, Norris SL, Akl EA, Schünemann HJ (2011). GRADE guidelines: 5. Rating the quality of evidence—publication bias. J Clin Epidemiol.

[29] Guyatt GH, Oxman AD, Kunz R, Woodcock J, Brozek J, Helfand M, Alonso-Coello P, Glasziou P, Jaeschke R, Akl EA, Norris S, Vist G, Dahm P, Shukla VK, Higgins J, Falck-Ytter Y, Schünemann HJ (2011). GRADE guidelines: 7. Rating the quality of evidence—inconsistency. J Clin Epidemiol.

[30] Guyatt GH, Oxman AD, Kunz R, Woodcock J, Brozek J, Helfand M, Alonso-Coello P, Falck-Ytter Y, Jaeschke R, Vist G, Akl EA, Post PN, Norris S, Meerpohl J, Shukla VK, Nasser M, Schünemann HJ, GRADE Working Group (2011). GRADE guidelines: 8. Rating the quality of evidence—indirectness. J Clin Epidemiol.

[31] Hussain-Gambles M, Atkin K, Leese B (2004). Why ethnic minority groups are under-represented in clinical trials: a review of the literature. Health & social care in the community.

[32] The Biden-Harris Plan to beat COVID-19 [Available from: https://buildbackbetter.com/priorities/covid-19/.

